# Piperlongumine-Eluting Gastrointestinal Stent Using Reactive Oxygen Species-Sensitive Nanofiber Mats for Inhibition of Cholangiocarcinoma Cells

**DOI:** 10.1186/s11671-019-2887-0

**Published:** 2019-02-18

**Authors:** Hyung Ha Jang, Su Bum Park, Jeong Sup Hong, Hye Lim Lee, Yeon Hui Song, Jungsoo Kim, Yun Hye Jung, Chan Kim, Doo-Man Kim, Sang Eun Lee, Young-Il Jeong, Dae Hwan Kang

**Affiliations:** 1 School of Medicine, Pusan National University, Yangsan, Gyeongnam 50612 South Korea; 2 Research Institute of Convergence of Biomedical Sciences, Pusan National University Yangsan Hospital, Yangsan, Gyeongnam 50612 South Korea; 3 Division of Animal Care, Yonam College, Cheonan, Chungnam 31005 South Korea; 4 Amotech Co. Ltd, Incheon, Gyeonggi-do South Korea; 5 Department of Photonics Engineering, Chonnam National University, Gwangju, 61186 South Korea

**Keywords:** Piperlongumine, Drug-eluting stent, Nanofiber, GI stent, Cholangiocarcinoma

## Abstract

**Background:**

The aim of this study is to fabricate drug-eluting gastrointestinal (GI) stent using reactive oxygen species (ROS)-sensitive nanofiber mats for treatment of cholangiocarcinoma (CCA) cell. A ROS-producing agent, piperlongumine (PL)-incorporated nanofiber mats were investigated for drug-eluting stent (DES) application.

**Methods:**

Selenocystamine-conjugated methoxy poly(ethylene glycol) (MePEG) was conjugated with poly(L-lactide) (PLA) to produce block copolymer (LEse block copolymer). Various ratios of poly(ε-caprolactone) (PCL) and LEse block copolymer were dissolved in organic solvent with PL, and then nanofiber mats were fabricated by electro-spinning techniques.

**Results:**

The higher amount of LEse in the blend of PCL/LEse resulted in the formation of granules while PCL alone showed fine nanofiber structure. Nanofiber mats composed of PCL/LEse polymer blend showed ROS-sensitive drug release, i.e., PL release rate from nanofiber mats was accelerated in the presence of hydrogen peroxide (H_2_O_2_) while nanofiber mats of PCL alone have small changes in drug release rate, indicating that PL-incorporated nanofiber membranes have ROS responsiveness. PL itself and PL released from nanofiber mats showed almost similar anticancer activity against various CCA cells. Furthermore, PL released from nanofiber mats properly produced ROS generation and induced apoptosis of CCA cells as well as PL itself. In HuCC-T1 cell-bearing mice, PL-incorporated nanofiber mats showed improvement in anticancer activity.

**Conclusion:**

PL-incorporated ROS-sensitive nanofiber mats were coated onto GI stent and showed improved anticancer activity with ROS responsiveness. We suggested PL-incorporated ROS-sensitive nanofiber mats as a promising candidate for local treatment of CCA cells.

## Background

Cholangiocarcinoma (CCA), which is normally derived from the bile duct region, is regarded as one of the most aggressive cancers [1–3]. The reason of carcinogenesis and increase in incidence rate are still unclear even though its incidence rate is increasing over worldwide [1]. Since most of the CCA patients are frequently diagnosed in advanced state, very few cases of the CCA patients are amenable to surgical resection [2]. Various treatment options such as radiotherapy, chemotherapy, metal stent displacement, and immune-adjuvant therapy have been tried to treat CCA patients [3–9]. Since bile duct is blocked by tumor growth or inflammation, stent displacement is frequently employed to prevent bile duct occlusion and to prolong patient survivability amongst aforementioned treatments [10, 11]. However, metal stent has no curative function, and tumor-overgrowth inside stent normally induces biliary obstruction. To solve these problems, many scientists have investigated drug-eluting stent (DES) [12–15]. For example, Lee group investigated paclitaxel-eluting gastrointestinal (GI) stent in the last decade [12–15]. Although paclitaxel-eluting stent has little differences in stent patency and patient survivability compared to a covered metal stent, they observed acceptability of paclitaxel-eluting stent in porcine feasibility and safety study [13–15]. Kim et al. investigated DES using sorafenib, a tyrosine protein kinase inhibitor, against HuCC-T1 CCA cells using animal tumor xenograft models, and sorafenib-eluting stent has efficacies to inhibit tumor growth in animal tumor xenograft model [16]. Kwak et al. reported that histone deacetylase (HDAC) inhibitor (vorinostat)-eluting stent effectively inhibits expression of HDAC, induces acetylated histone (Ac-histone), and then inhibits tumor growth in CCA cell-bearing mice model [17]. DES with novel anticancer agent or molecular target agent is still an attractive option to prolong patient survivability. Furthermore, stimuli-sensitive nanofiber mats or nanomaterials also have been specifically investigated to deliver the anticancer agent [18–20]. Nanofibers based on thermosensitive polymers such as poly(di(ethylene glycol) methyl ether methacrylate) (PDEGMA) or poly(*N*-isopropylacrylamide) poly(NIPAM) copolymers can be applicable in temperature-sensitive drug release in local disease site [18, 19]. Bellat et al. reported that self-assembling peptide nanofibers were shown in excellent tumor targeting with reduced RES capture [20].

Imbalance between the production of ROS and cellular antioxidant defense system in disease has been extensively investigated in the biomedical field [21–24]. Especially, oxidative stress in cancers has been also extensively investigated and applied in redox-sensitive drug delivery system [23–25]. For example, thioether groups in the polymer backbone of the nanoparticles can be changed from hydrophobic to hydrophilic, when it was exposed to ROS, and ROS-switchable nanoparticles can be applicable in disease with ROS-rich environment [25, 26]. Yu et al. reported that specific endosomal delivery into cancer cells can be achieved by ROS-responsive polymer micelles, i.e., copolymer can be converted from hydrophobic to hydrophilic in ROS-rich environment, and this phenomenon induced rapid release of anticancer drug following higher anticancer activity [27]. We also previously reported that redox-responsive nanophotosensitizers specifically release photosensitizer with glutathione (GSH)-responsiveness and then induced higher ROS generation compared to photosensitizer itself [28]. In recent studies, polymer micelles having diselenide linkage is known to have ROS-triggered drug release through the disintegration of diselenium linkages and to show ROS-specific anticancer activity [29]. ROS-triggered nanoparticles are regarded as a promising platform for anticancer chemotherapy.

Piperlongumine (PL), a natural chemical originated from *Piper longum*, has promising anticancer activities with low cytotoxicity against normal cells [30]. Raj et al. reported that cancer cell-selective toxicity of PL is due to the fact that PL selectively produces ROS in cancer cells relative to normal cell; PL induces DNA damage and alterations in mitochondria morphology/function in cancer cells [30]. Anticancer activity of PL against various cancer cells has been investigated [31–35]. Xiong et al. reported that PL markedly increased ROS and then effectively inhibited primary myeloid leukemia cells through induction of apoptotic proteins [35]. Even though PL has low toxicity against normal cells and organs, it has some adverse effects on the kidney [36, 37]. Furthermore, short half-life of PL in the bloodstream has also to be improved [38].

In this study, we fabricated a redox-responsive nanofiber-coated stent and PL was loaded in the nanofiber mats. For redox-responsiveness, LEse block copolymer having diselenide linkage was synthesized and used to fabricate nanofiber mats for DES. The increased ROS contents in the tumor region may accelerate drug release rate and promote ROS-mediated cancer cell death.

## Materials and Methods

### Materials

PL was purchased from LKT Labs. Co., (Minnesota, USA). Poly(L-lactide) (PLA, PLA-0005, M.W. = 5000 g/mol from the manufacturer’s data) was purchased from Wako Pure Chem. Co. Ltd. (Osaka, Japan). Methoxy poly(ethylene lycol)-succinimidylglutarate (MePEG-NHS, M.W. = 5000 g/mol) was purchased from Sunbio Co. Ltd. (Seoul, Korea). Silicon-membrane covered stent for biliary tract was obtained from M.I. Tech. (Pyeongtaek, Korea). Poly(ε-caprolactone) (PCL, number-average M.W. = 80,000 g/mol), N-hydroxysuccinimide (NHS), N-(3-dimethylaminopropyl)-N′-ethylcarbodiimide hydrochloride (EDAC), tetrahydrofuran (THF), chloroform, dimethyl sulfoxide (DMSO), 3-(4,5-dimethylthiazol-2-yl)-2,5-diphenyltetrazolium bromide (MTT), and selenocystamine dihydrochloride were purchased from Sigma-Aldrich Chemical Co., (St. Louis, MO, USA). The dialysis membrane or dialysis device (M.W. cutoff size (MWCO) 1000 g/mol and 8000 g/mol) was purchased from Spectrum/Por Lab., Inc. (CA, USA). All organic solvents and other chemicals were used as extra-pure grade. Cell culture supplies such as RPMI1640 media and fetal bovine serum (FBS) were purchased from Life Tech. Inc. (Grand Island, NY, USA). All reagent and organic solvents used were HPLC-grade.

### Synthesis of LEse Block Copolymer

#### MePEG-selenocystamine conjugates

MePEG-NHS (500 mg) was dissolved in 20 mL DMSO. More than five equivalents of selenocystamine were separately dissolved in 15 mL deionized water and mixed with 5 mL DMSO. After that, selenocystamine solution was slowly dropped to MePEG-NHS solution and then magnetically stirred for 24 h. Following this, reactants were introduced into a dialysis tube (MWCO 1000 g/mol) and then dialyzed against plenty of water for 2 days. Dialyzed solution was lyophilized for 2 days. Lyophilized solid was used to synthesize block copolymer.

#### Block copolymer synthesis

PLA (500 mg) was dissolved in 20 mL DMSO with equivalent amount of EDAC and NHS. This solution was magnetically stirred for 12 h. To this solution, mPEG-selenocystamine solid (530 mg) was added and further stirred for 2 days. Following this, reactants were introduced into a dialysis tube (MWCO: 8000 g/mol) and then dialyzed against plenty of water for 2 days. Dialyzed solution was lyophilized for 2 days. Lyophilized products were precipitated in methanol to remove unreacted mPEG-selenocystamine once more. The final yield was higher than 94%. Yield = [(weight of lyophilized solid)/(weight of PLA + weight of mPEG-selenocystamine)] × 100.

### Characterization of Polymers

To monitor polymer synthesis, ^1^H-nuclear magnetic resonance (NMR) spectroscopy was employed. Polymers were dissolved in DMSO-d form and measured with ^1^H-NMR spectroscopy (500 MHz Superconducting FT-NMR Spectrometer, UnityInova 500, Varian Inc. Agilent Tech., CA, USA).

Molecular weight of polymers was measured with gel-permeation chromatography (GPC, Waters GPC system, MA 01757, USA): Waters 1515 HPLC solvent pump, a Waters 2414 refractive index detector, and three Waters Styragel High Resolution columns (HR4, HR2, HR1). Polymers were dissolved in extra pure-grade THF containing 0.1 N LiBr as eluent (flow rate 1.0 mL/min). Polystyrenes were used to make a calibration curve.

### PL-Loaded Nanofiber Mats and Coated on the GI Stent

#### PL-loaded nanofiber mats

Vorinostat (100 mg) was dissolved in 10 mL acetone solution. To this solution, LEse (100~400 mg) and PCL (600~900 mg) was then added and magnetically stirred for 2 h. This solution was used to fabricate nanofiber mats and to coat onto the silicone membrane-covered stent using an electrospinning machine (EBS ES-Biocoater; Nano NC, Seoul, South Korea). Electrospinning machine is composed of a high-voltage power supply, syringe pump, X-Y robotic system, and drum-roll collector. The polymer/drug solution in a syringe (NanoNC, 24G) was sprayed onto the silicone membrane-covered metal stent (diameter 1 cm, length 10 cm, rolling speed 500 rpm, spray rate 100 μL/minute, voltage 15 kV) which is placed onto the rolling collector. PL-loaded nanofiber membrane was coated onto the stent. To remove remaining solvent, PL-loaded nanofiber coated stent was dried in a vacuum drying oven over 24 h. PL-loaded nanofiber coated stents were stored at 4 °C until the following study.

PL-loaded nanofiber mats were carefully separated from the stent for drug release and animal study. Empty nanofiber membrane was prepared in the absence of PL with a similar procedure.

Drug contents were measured as follows: 5 mg of PL-incorporated nanofiber mats was dissolved in DMSO for 2 h. UV-spectrophotometer (UV-1601, Shimadzu Co. Ltd. Osaka, Japan) was used to measure drug concentration at 325 nm. Empty nanofiber mats were also dissolved in DMSO and used for blank test.

$$ \mathrm{Drug}\ \mathrm{content}\ \left(\%,w/w\right)=\left(\mathrm{PL}\ \mathrm{weight}/\mathrm{total}\ \mathrm{weight}\ \mathrm{of}\ \mathrm{nanofiber}\ \mathrm{mats}\right)\times 100. $$

Drug release study was performed with phosphate-buffered saline (PBS, 0.01 M, pH 7.4) in vitro. Nanofiber mats were cut into disks, and 10 mg of disks was immersed into 40 mL PBS (0.01 M, pH 7.4) in a 50 mL conical tube. This was placed into a shaking incubator at 100 rpm (37 °C). Whole media were taken to measure the concentration of released PL from nanofiber mats at specific time intervals. The PL concentration in the media was measured with a UV-spectrophotometer (325 nm). Empty nanofiber mats were also employed to use as a blank test. In the release study, hydrogen peroxide was added to the release media to investigate ROS effect on the drug release rate.

### Morphology

Morphology of nanofiber mats was observed with a field-emission scanning electron microscope (S-4800; Hitachi, Tokyo, Japan) at 25 kV.

### Cell Culture

Human CCA cell lines such as SNU478, SNU245, and SNU 1196 CCA cell lines were obtained from the Korean Cell Line Bank (Seoul, Korea). HuCC-T1 human CCA cell line was obtained from the Health Science Research Resources Bank (Osaka, Japan). All the cells were cultured in RPMI1640 supplemented with 10% heat-inactivated FBS and 1% penicillin/streptomycin at 37 °C in a 5% CO_2_ incubator.

### Anticancer Activity Study

Various CCA cells (1 × 10^4^) seeded in 96-well plates were used to evaluate anticancer activity of PL itself, PL-incorporated nanofiber mats, or PL released from nanofiber mats. Cells were maintained overnight in a 5% CO_2_ incubator at 37 °C. For PL treatment, PL dissolved in DMSO (10 mg PL/mL DMSO) were diluted with RPMI1640 media. The final concentration of DMSO was lower than 0.5%. For treatment of PL released from nanofiber mats, PL-incorporated nanofiber mats were immersed in 40 mL PBS in 50 mL conical tube. On day 5 and day 15, PL concentration was measured with UV-spectrophotometer as described above and used to treat cells. Empty nanofiber was also adapted to the release experiment for comparison. Cells were exposed to PL itself or PL released from nanofiber mats for 2 days. The viability of CCA cells was evaluated with MTT proliferation assay. Thirty-microliter MTT solution (5 mg/mL PBS, pH 7.4) was added to the wells, and then the cells were incubated for 4 h in a 5% CO_2_ incubator at 37 °C. The medium was discarded and added 100 μl DMSO. Cell viability was analyzed with a microplate reader at 570 nm (Infinite M200 Pro microplate reader, Tecan, Mannedorf, Switzerland). Cell viability was expressed as mean ± standard deviation from eight wells.

### ROS Measurement

ROS generation in CCA cells was assayed by DCFH-DA method. CCA cells (1 × 10^4^ cells) seeded in a 96-well plate were treated with various concentrations of PL itself or PL released from nanofiber mats in phenol red-free RPMI media with DCFH-DA (final concentration 20 μM). Six hours or 12 h later, cells were washed with PBS twice and replaced with 100 μL fresh phenol red-free RPMI media. ROS contents in cells were analyzed by fluorescence intensity changes using the Infinite M200 pro microplate reader (Excitation wavelength 485 nm, emission wavelength 535 nm).

### Western Blotting

Western blotting of CCA cells was carried out as previously described [31]. Cells were exposed to PL itself or PL released from nanofiber mats for 24 h. After that, cells were harvested by trypsinization, washed with cold PBS, and collected by centrifugation. Pellets were lysed in lysis buffer containing 50 mM Tris, 150 mM NaCl, 1% NP-40, 0.5% deoxycholic acid, 0.1% sodium dodecyl sulfate (SDS) with phenylmethylsulfonyl fluoride, and a protease inhibitor cocktail (Roche Diagnostics, Basel, Switzerland). This solution was centrifuged for 30 min at 4 °C (14,000×*g*); the cell lysates (supernatant) were then used to measure protein concentration using the BCA Protein Assay kit (Pierce, Rockford, IL, USA). Protein (50 μg) was loaded into SDS-polyacrylamide gel electrophoresis (SDS-PAGE), transferred to a polyvinyl difluoride (PVDF) membrane, blocked with 5% skim milk in TBS-T, probed with an appropriate primary antibody, and then treated with a secondary HRP-conjugated antibody for 1 h. The immunoblots were detected by chemiluminescence and then quantified with digital analyses using the ImageJ software program.

### In Vivo Study Using Tumor Xenograft Model

HuCC-T1-bearing mice (BALB/c nude mouse, 5 weeks old, male, 18–23 g in weight; Orient, Seongnam, South Korea) were used to evaluate in vivo antitumor activity of PL-incorporated nanofiber stent. 1 × 10^6^ HuCC-T1 cells in 100 μL of PBS were administered subcutaneously (s.c.) to the backs of nude mice. Disks of PL-incorporated nanofiber and empty nanofiber were implanted under the solid tumor when the solid tumor became approximately 4~5 mm diameter. The dose of PL was 10 mg PL/kg. For comparison, PL was dissolved in Cremophor EL®/ethanol mixture solution (0.5% *v*/*v* Cremophor EL® and 0.5% *v*/*v* ethanol in PBS (pH 7.4, 0.01 M)). Control groups were subcutaneously injected with PBS beside the tumor tissue. For PL-incorporated nanofiber and empty nanofiber group, nanofiber disks were prepared as follows; nanofiber wafers with same weight were cut into round disks and then the back of the mouse’s skin was carefully excised (0.5 cm in length). Following this, nanofiber wafers were carefully implanted under the solid tumor tissue. To make an equal condition, mice with control treatment and PL injection have also excised skin beside the tumor (0.5 cm in length). Each group consisted of five mice. Tumor volume was measured with intervals of 5 days, and the first day of nanofiber implantation was set as day 0. Tumor volume was calculated by the following equation: *V* = (*a* × [*b*]^2^)/2. *a*: largest diameter; *b*: smallest diameter.

All the animal study was carefully performed under the guidelines of the Pusan National University Institutional Animal Care and Use Committee (PNUIACUC). The animal protocol used in this study has been strictly reviewed by the PNUIACUC on their ethical procedures and scientific care, and it has been approved (Approval Number: PNU-2017-1608).

### Immunohistochemistry

Tumor tissues were isolated 30 days later. Then, tumor tissues were fixed in 4% formaldehyde, paraffin-embedded, and sliced for hematoxylin and eosin (H&E) staining. Immunohistochemical staining was performed with apoptosis-related proteins such as caspase-3 and caspase-9 antibodies. Antibodies were used at a dilution of 1:100 or 1:200, and then staining was performed using an Envision kit (Life Technologies, Carlsbad, CA, USA) according to the manufacturer’s protocol.

### Statistical Analysis

Statistical analyses of the data from treated and untreated cells were performed using the Student’s *t* test. A *p* value < 0.05 was considered to be statistically significant.

## Results

### Characterization of Polymers

**Fig. 1:**
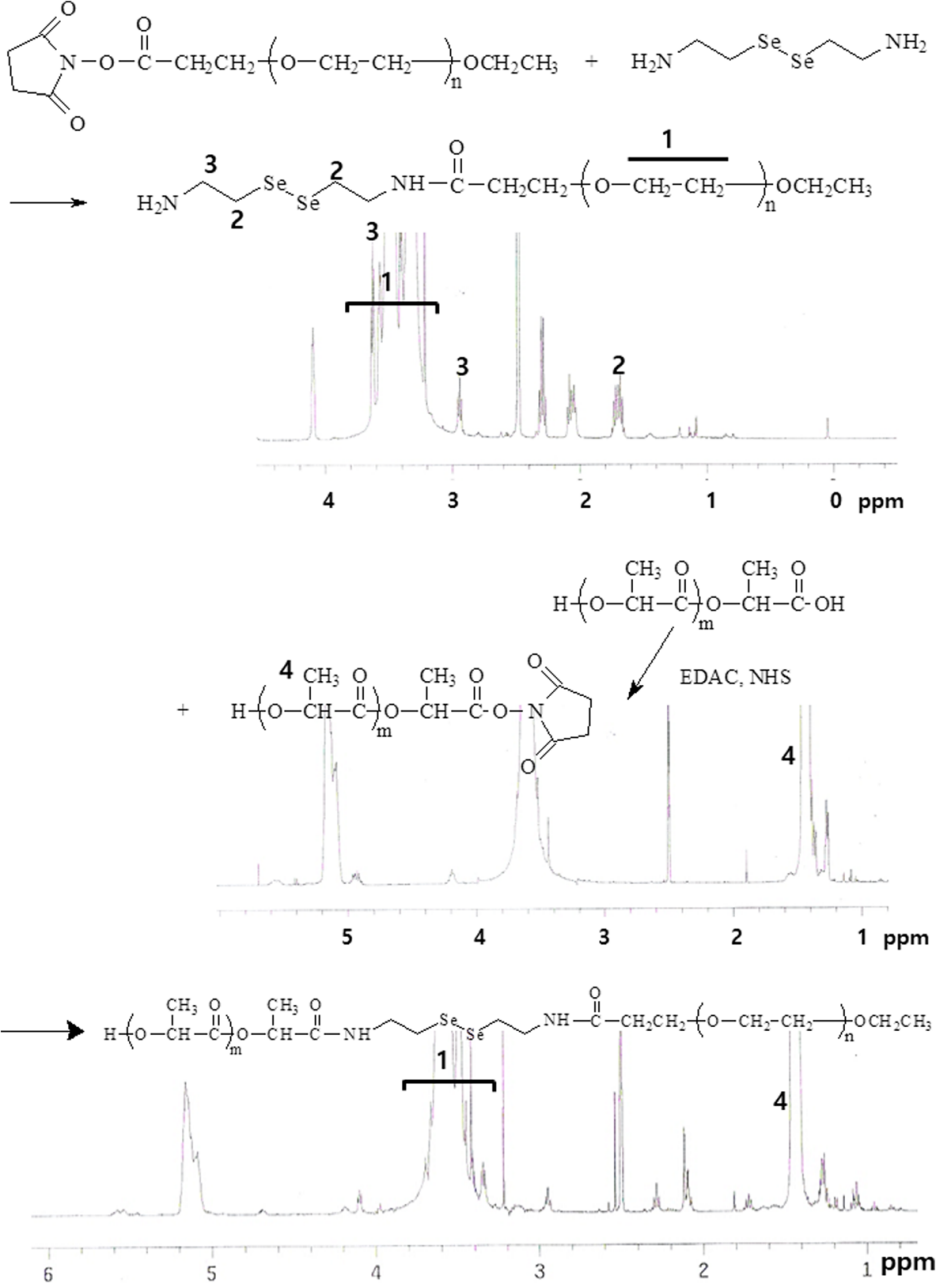
Synthesis scheme of LEse block copolymer

To fabricate PL-eluting GI stent, LEse block copolymer was synthesized as shown in Fig. 1. MePEG-NHS was reacted with selenocystamine, and then the terminal amine group was conjugated with the carboxyl end group of PLA. Unreacted selenocystamine from MePEG-selenocystamine conjugates was removed by dialysis procedure. Furthermore, unreacted MePEG-selenocystamine conjugates from synthesized block copolymer were also removed by dialysis procedure and precipitation in methanol. Specific peaks of selenocystamine were confirmed at 1.7 ppm and 2.9 ppm, respectively, while the specific peak of MePEG was also confirmed at 3.5~3.7 ppm. When PLA was conjugated, the methyl group of PLA was confirmed at 1.4 ppm. PCL homopolymer and LEse block copolymer blend were blended to fabricate nanofiber mats. M.W. and composition of LEse block copolymer and PCL homopolymer were measured with ^1^H-NMR spectroscopy and GPC. The results of M.W. estimation was shown in Table 1. As shown in Table 1, M.W. of LEse block copolymer was estimated based on the M.W. of PEG using ^1^H-NMR spectroscopy as 9760 g/mol. GPC measurement showed that LEse block copolymer has 8210 g/mol of Mn, 9530 g/mol of Mw, and 1.16, respectively.

**Table 1:** Characterization of polymers

	M.W. by GPC			M.W. calculated by ^1^H-NMR spectroscopy ^a^
	Mn	Mw	PD	
Me-PEG 5 k	4390	5160	1.18	5000^a^
PLA	4120	4890	1.19	4530^b^
LEse copolymer	8210	9530	1.16	9530
PCL	72,100	81,200	1.13	–

^a^MePEG-5 k: MePEG-NHS, M.W. = 5000 g/mol from the manufacturer. M.W. estimation of block copolymer using ^1^H-NMR spectroscopy was calculated based on MePEG-5 k

^b^M.W. of PLA was calculated from following the equation M.W. of LEse copolymer – M.W. of MePEG 5 k

*Mn* number-average M.W, *Mw* weight average M.W, *PD* polydispersity

### Characterization of Piperlongumine-Incorporated Nanofiber Coated GI Stent

**Fig. 2:**
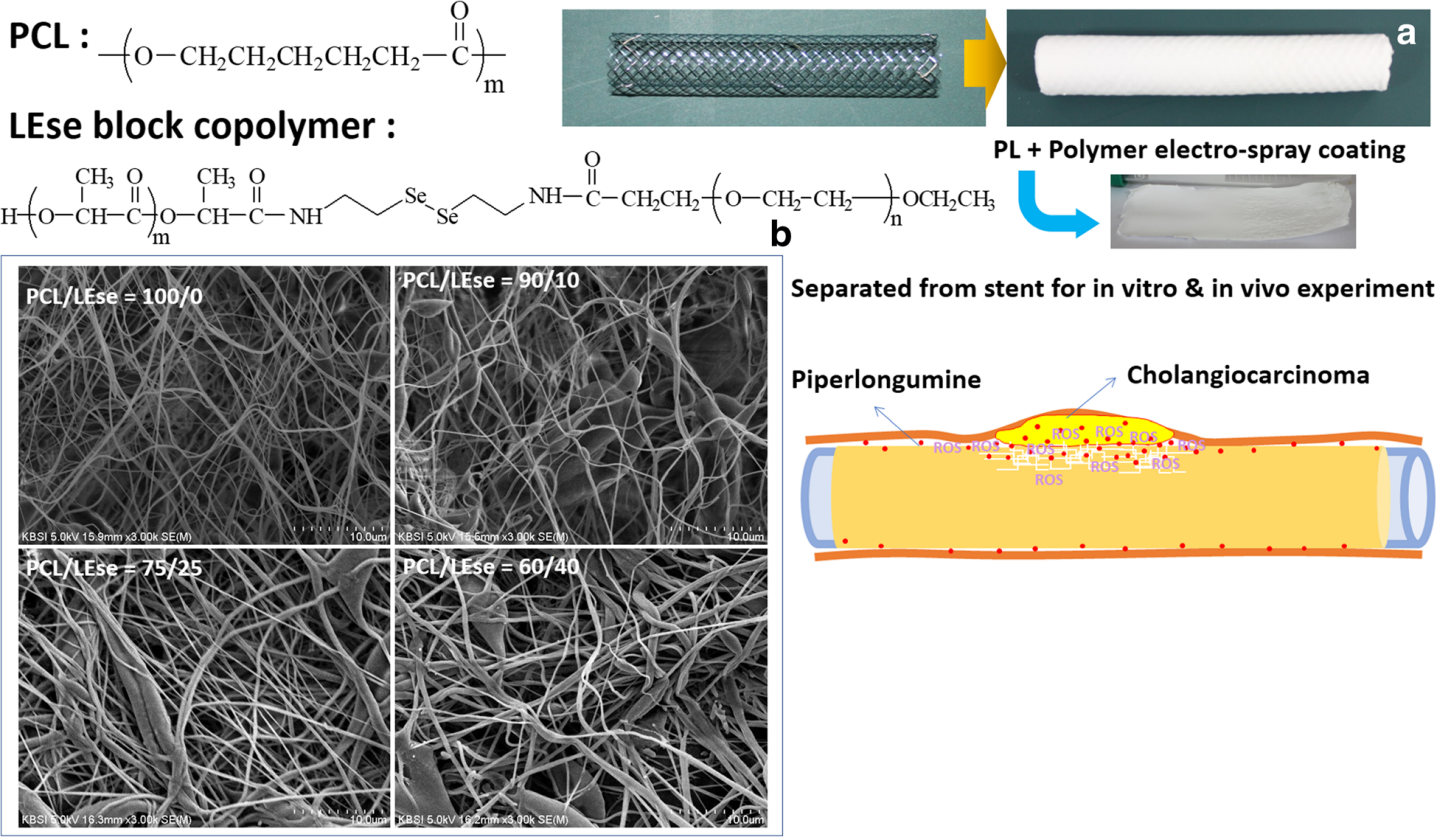
**a** PL-incorporated nanofiber-covered GI stent. **b** FE-SEM photo of PL-incorporated nanofiber

As shown in Fig. 2 and Table 2, various ratios of PCL and LEse block copolymer were used to fabricate nanofiber and to coat onto GI stent. PCL homopolymer resulted in fine and thin nanofiber mats with minimized aggregated form. When LEse block copolymer was added, some of the aggregated form such as granules and particles was observed as shown in Fig. 2. At higher LEse ratio (75/25 and 60/40), nanofiber mats displayed a thicker and irregular form of fibrous structure. When consisted of more than 50% ratio of LEse in their contents, polymers were significantly aggregated and mats showed severe irregularity (data not shown). Nanofibrous structure was hardly obtained from LEse block copolymer alone. Therefore, nanofibrous structure can be attained by blending with PCL homopolymer. Drug contents in prepared PL-incorporated nanofiber mats were almost similar to theoretical value as shown in Table 2. These results indicated that PL-incorporated nanofiber mats were successfully fabricated from PCL homopolymer and LEse block copolymer mixtures and then coated onto GI stent.

**Table 2:** Characterization of PL-incorporated nanofiber mats

PCL/LEse weight ratio (mg/mg)	Drug content (%, *w*/*w*)	
	Theoretical	Experimental
1000/0	9.1	9.1 ± 0.1
900/100	9.1	9.1 ± 0.1
750/250	9.1	9.0 ± 0.12
600/400	9.1	8.9 ± 0.11

**Fig. 3:**
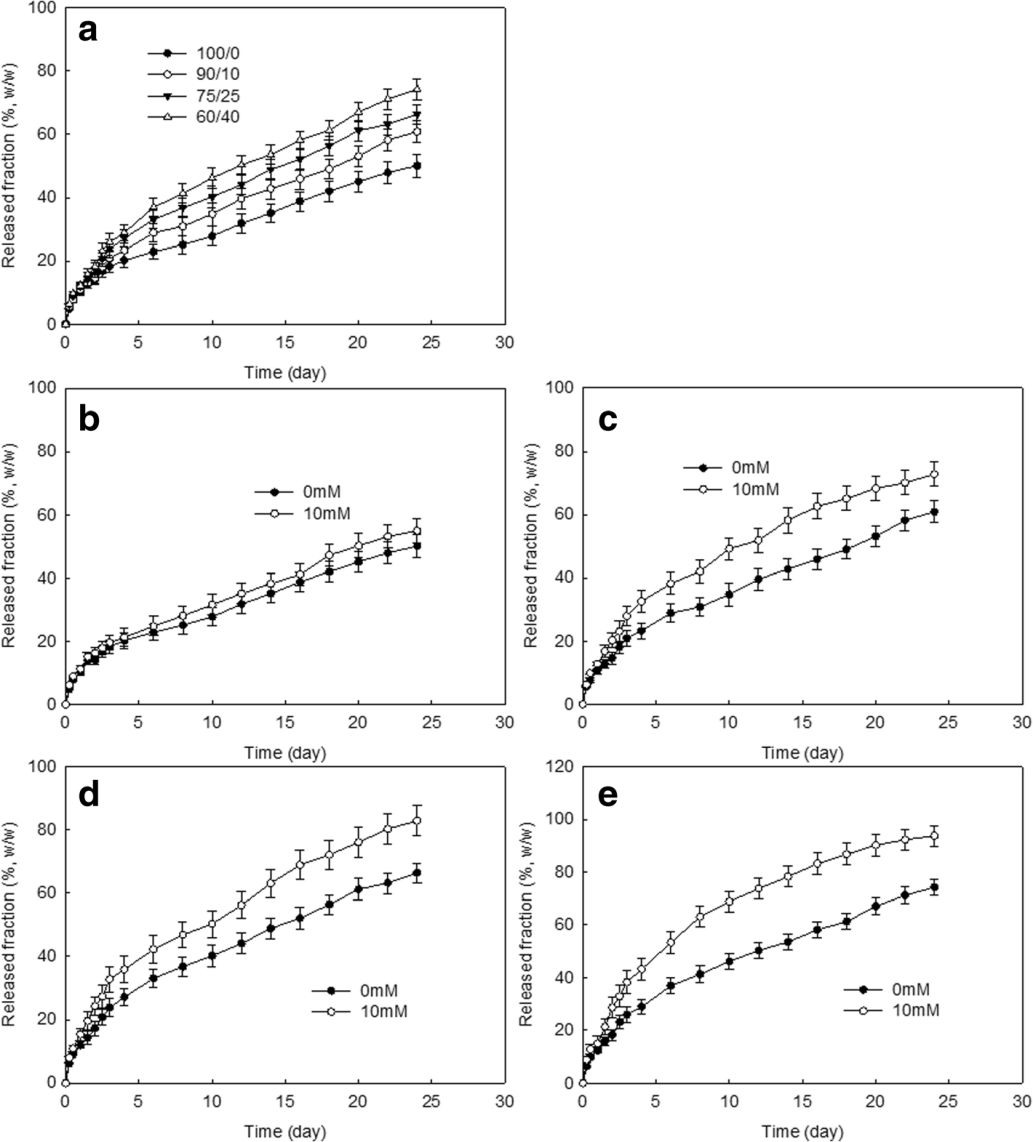
**a** PL release from nanofibers having various compositions. PCL/Lese weight ratio was 100/0, 90/10, 75/25, and 60/40, respectively. **b** The effect of hydrogen peroxide on the PL release from nanofiber mats (PCL/Lese weight ratio was 100/0). **c** The effect of hydrogen peroxide on the PL release from nanofiber mats (PCL/Lese weight ratio was 90/10). **d** The effect of hydrogen peroxide on the PL release from nanofiber mats (PCL/Lese weight ratio was 75/25). **e** The effect of hydrogen peroxide on the PL release from nanofiber mats (PCL/Lese weight ratio was 60/40)

Fig. 3 shows drug release kinetics from nanofiber mats. As shown in Fig. 3a, PL was continuously released from nanofiber mats over 25 days. Burst release of PL from nanofiber mats was observed until 4 days, and then PL was continuously released from nanofiber mats until day 25. Higher contents of LEse block copolymer in nanofiber mats resulted in faster release of PL from nanofiber mats. Since LEse block copolymer is less hydrophobic than PCL homopolymer, PCL/LEse nanofiber mats with higher content of LEse block copolymer must be swelled more than that of PCL homopolymer. Then, nanofiber mats with higher content of LEse block copolymer resulted in faster drug release. Furthermore, PL release rate can be accelerated in the presence of ROS since diselenide linkage in LEse block copolymer can be disintegrated by ROS such as H_2_O_2_, and then these factors induce redox-responsive disintegration of nanofiber mats (Fig. 3c~e). Nanofiber mats of PCL homopolymer were not significantly responsive to the addition of H_2_O_2_, i.e., PCL is not largely affected by H_2_O_2_addition; on the other hand, when LEse block copolymer was blended in, PL release from nanofiber mats was significantly accelerated as H_2_O_2_ was added . Especially, higher LEse block copolymer ratio in the nanofiber mats induced faster drug release kinetics as shown in Fig. 3c, d, and e. These results indicated that PL-incorporated nanofiber mats have redox-responsive drug release potential in the biological environment. PL-incorporated nanofiber mats prepared with PCL/LEse block copolymer (60/40) were used following in vitro cell culture and in vivo animal study.

### In Vitro Anticancer Activity

**Fig. 4:**
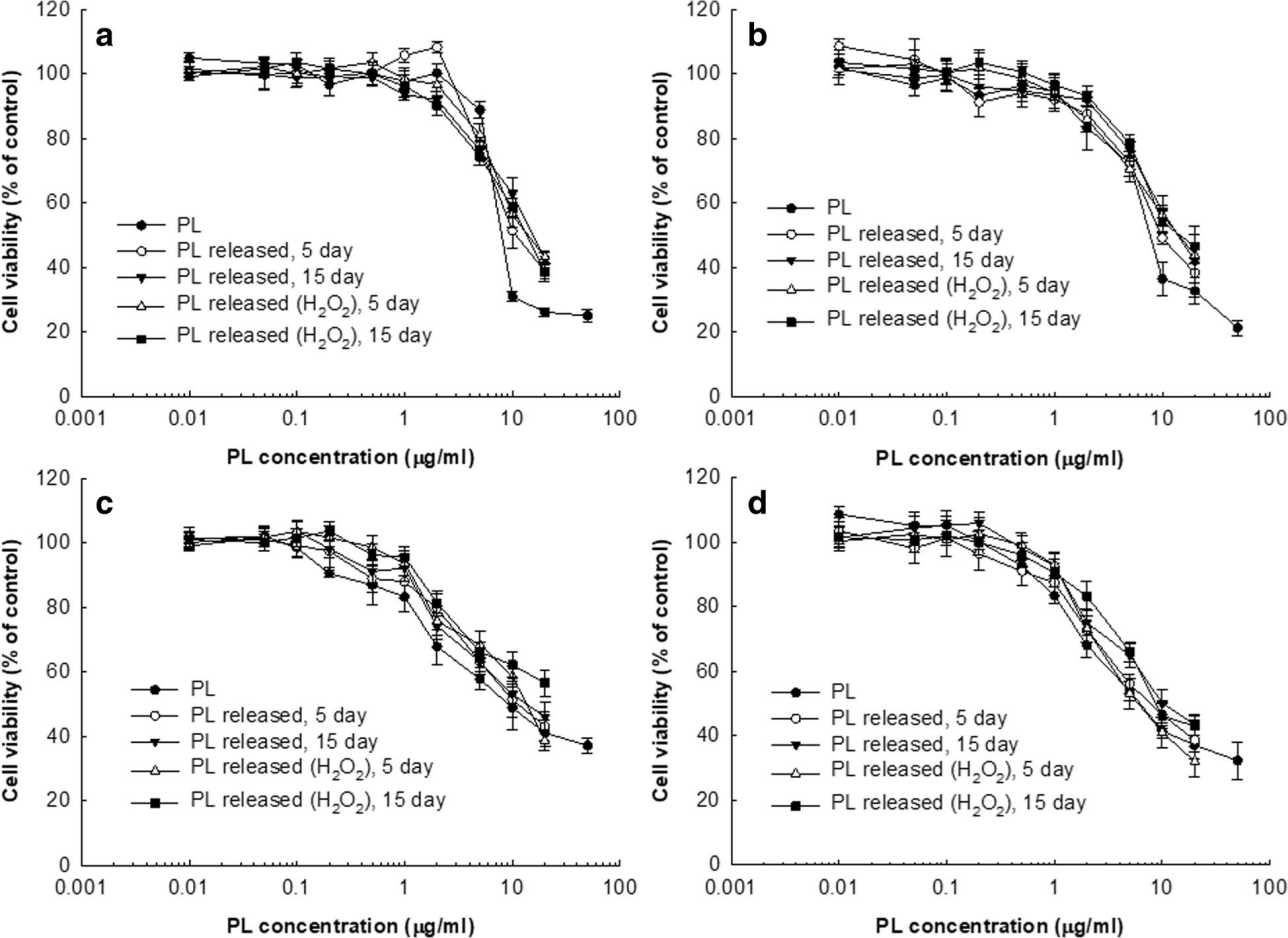
The effect of PL and released PL from nanofiber mats on the viability of various CCA cells. **a** HuCC-T1, **b** SNU1196, **c** SNU478, and **d** SNU245 cholangiocarcinoma cells. 2 × 10^4^ cells in 96-well plates were exposed to PL or released PL from nanofibers for 2 days. For treatment of released PL, PL-incorporated nanofiber disks were immersed into PBS and then release experiment was performed for 5 and 15 days with or without 10 mM H_2_O_2_. The medium was exchanged until 3 days as similar with drug release study. After that, the medium was harvested between 5 and 15 days of release experiment. This solution was used to investigate the comparison of anticancer activity of PL itself and released PL

**Fig. 5:**
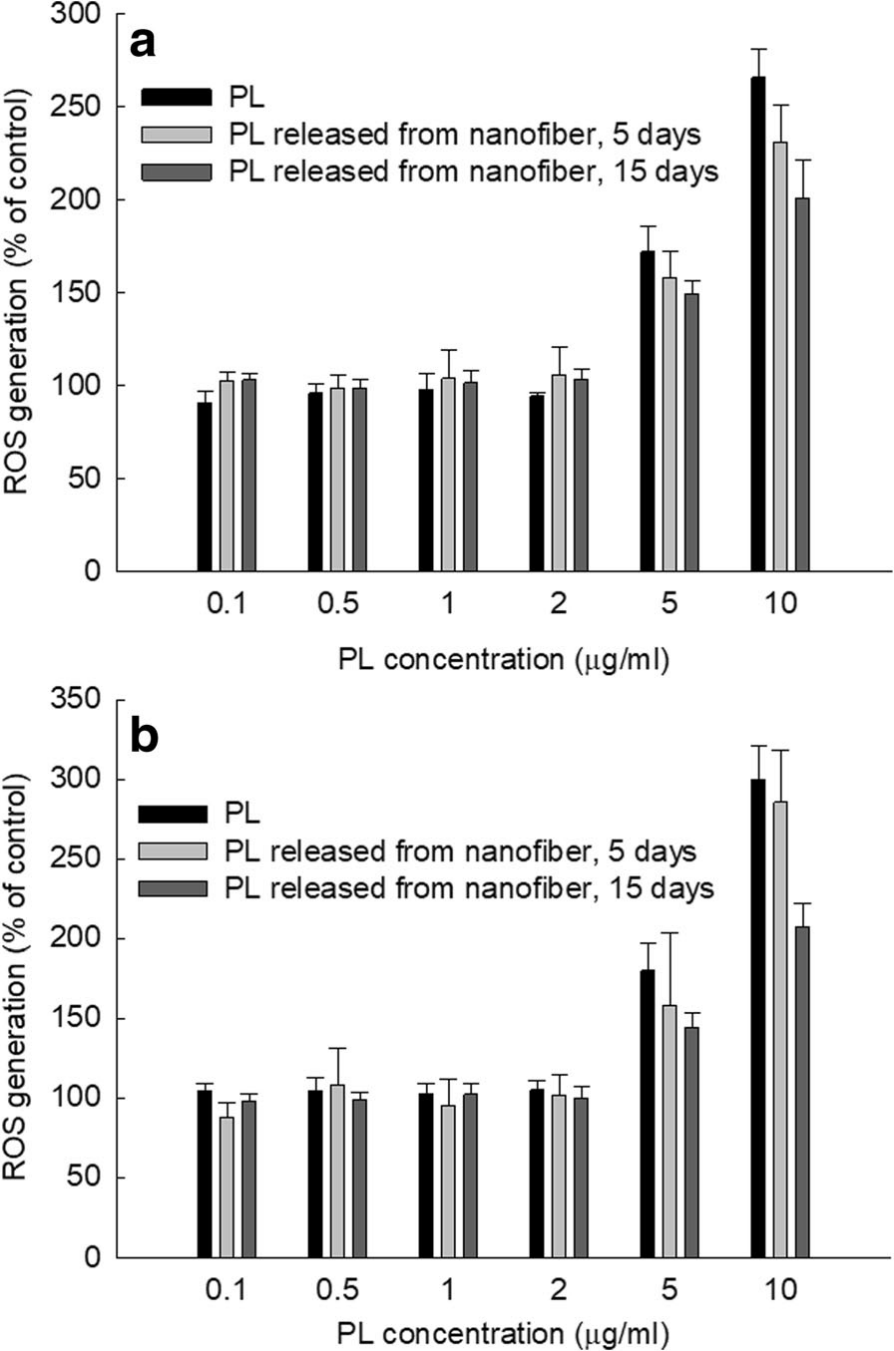
The effect of PL and released PL from nanofiber mats on the ROS generation of HuCC-T1 cells (**a**) and SNU245 cells (**b**). PL itself and released PL were treated as described in Fig. 3

Anticancer properties of PL-incorporated nanofiber mat-coated stent were assessed with various CCA cells. For comparison, PL released from nanofiber mats was extracted on day 5 and day 15 during the release experiment and was compared to intact PL. As shown in Fig. 4, anticancer activity on released PL from day 5 and day 15 did not significantly change compared to PL itself at all of HuCC-T1 cells (Fig. 4a), SNU1196 cells (Fig. 4b), SNU478 cells (Fig. 4c), and SNU245 cells (Fig. 4d). They have almost similar inhibition potential in cell viability even though PL itself resulted in higher anticancer activity at higher than 10 μg/mL concentration. Table 3 shows the IC_50_ value of PL itself and released PL from nanofiber mats. As well as PL itself, released PL from day 5 and day 15 maintained anticancer activity until 15 days of drug release experiment and showed reasonable IC_50_ value at all CCA cell lines although those values were gradually increased at PL from day 5 and day 15 compared to PL itself. Released PL from day 4 and day 15 in the presence of H_2_O_2_ also maintained anticancer activity as well as PL itself. These results indicated that anticancer activity of PL maintained during nanofiber fabrication process and drug release period in the biological environment. Furthermore, released PL from nanofiber mats produced ROS generation capacity until 15 days of drug release period as similar with PL itself (Fig. 5). As shown in Fig. 5a and b, PL itself produced ROS significantly more at higher rate than 5 μg/mL. Released PL from day 5 and day 15 also produced ROS although ROS production was slightly decreased by released PL from day 15, indicating that PL-incorporated nanofiber mats maintained intrinsic anticancer property of PL during drug release period.

**Table 3:** IC_50_ of PL itself and released PL from nanofiber mats against various CCA cells

	IC_50_ (μg/mL)^a^				
	PL itself	PL released from nanofiber		PL released from nanofibers in the presence of H_2_O_2_	
		Day 5	Day 15	Day 5	Day 15
HuCC-T1	8.3	11.0	16.3	14.8	14.1
SNU1196	8.1	9.8	14.7	14.9	15.5
SNU478	9.2	11.3	14.1	14.2	–^b^
SNU245	6.4	8.3	10.3	6.5	9.1

^a^ IC_50_ values of PL released from nanofiber were estimated from cell viability curve in Fig. 4

^b^ -, not determined

**Fig. 6:**
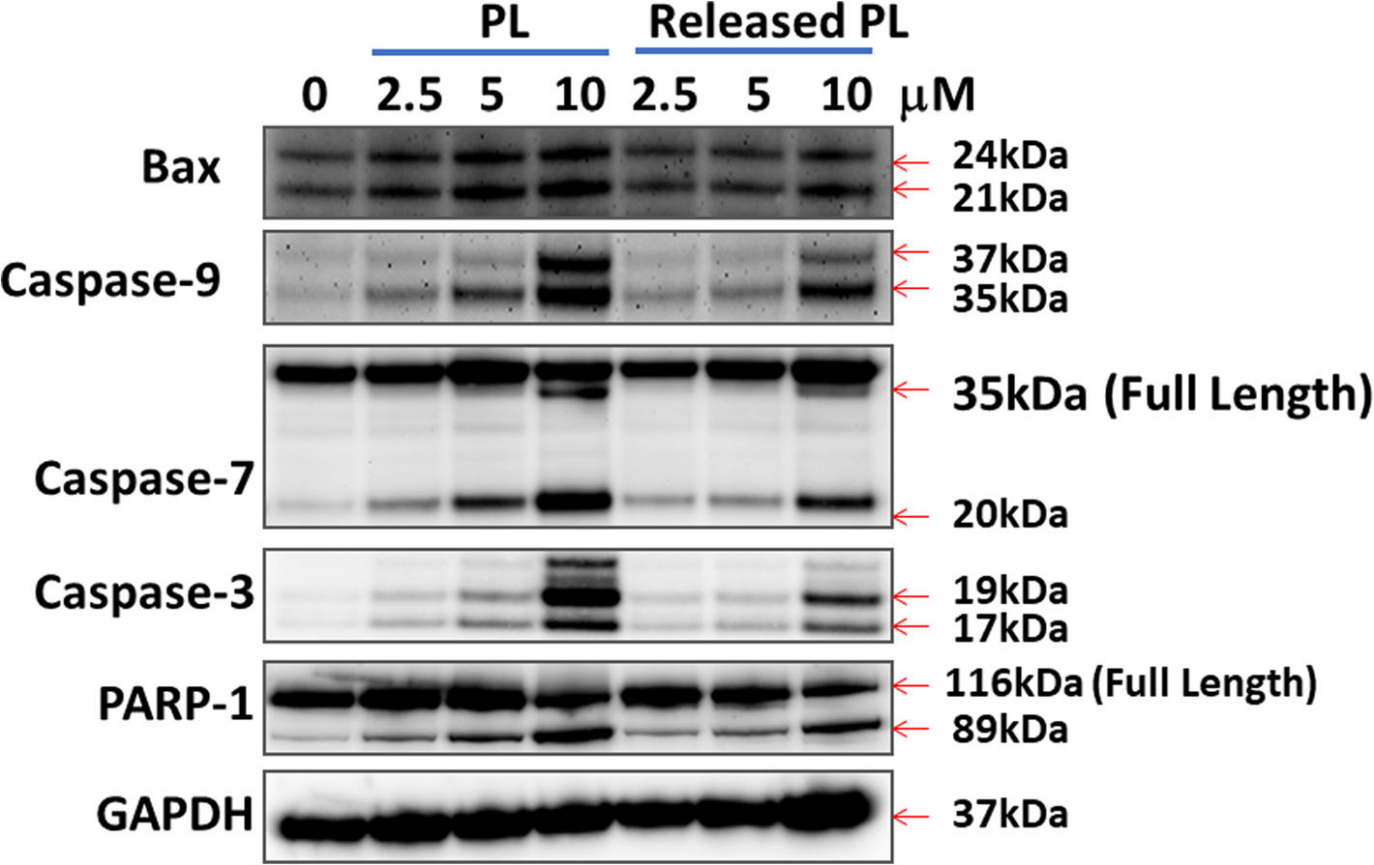
Western blot analysis of apoptosis of HuCC-T1 cells. Cells were treated PL itself or released PL from nanofibers for 1 day and then performed western blot analysis

Figure 6 showed the expression of apoptosis protein in HuCC-T1 CCA cells. Released PL (5 days) has similar activity in the induction of apoptosis of HuCC-T1 cells compared to PL itself, i.e., the expression of BAX, caspase-3, 7, and 9, and cleaved PARP cleavage was gradually increased by treatment of released PL (5 days) as well as PL itself. These results also indicated that released PL has reasonable anticancer activity against CCA cells as well as PL itself.

### In Vivo Anticancer Activity Against HuCC-T1 Tumor Xenograft Model

**Fig. 7:**
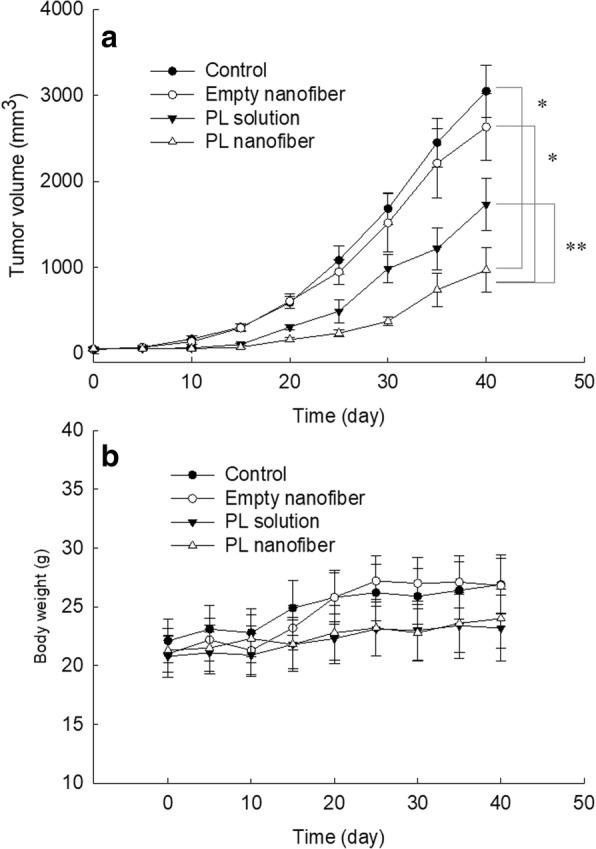
The effect of PL solution, empty nanofiber mats or PL-incorporated nanofiber mats on the growth of HuCC-T1 tumor (**a**) and the body weight changes (**b**). HuCC-T1 (1 × 10^6^ cells) cells were implanted to the back of mice. PL dose was adjusted to 10 mg/kg. One hundred microliters of PBS or PL solution was s.c. injected beside the tumor tissue for control treatment and PL solution treatment, respectively. For empty nanofiber and vorinostat nanofiber implantation, wafers of the same weight were cut and then implanted under the tumor tissue. *p < 0.001; **p < 0.01

**Fig. 8:**
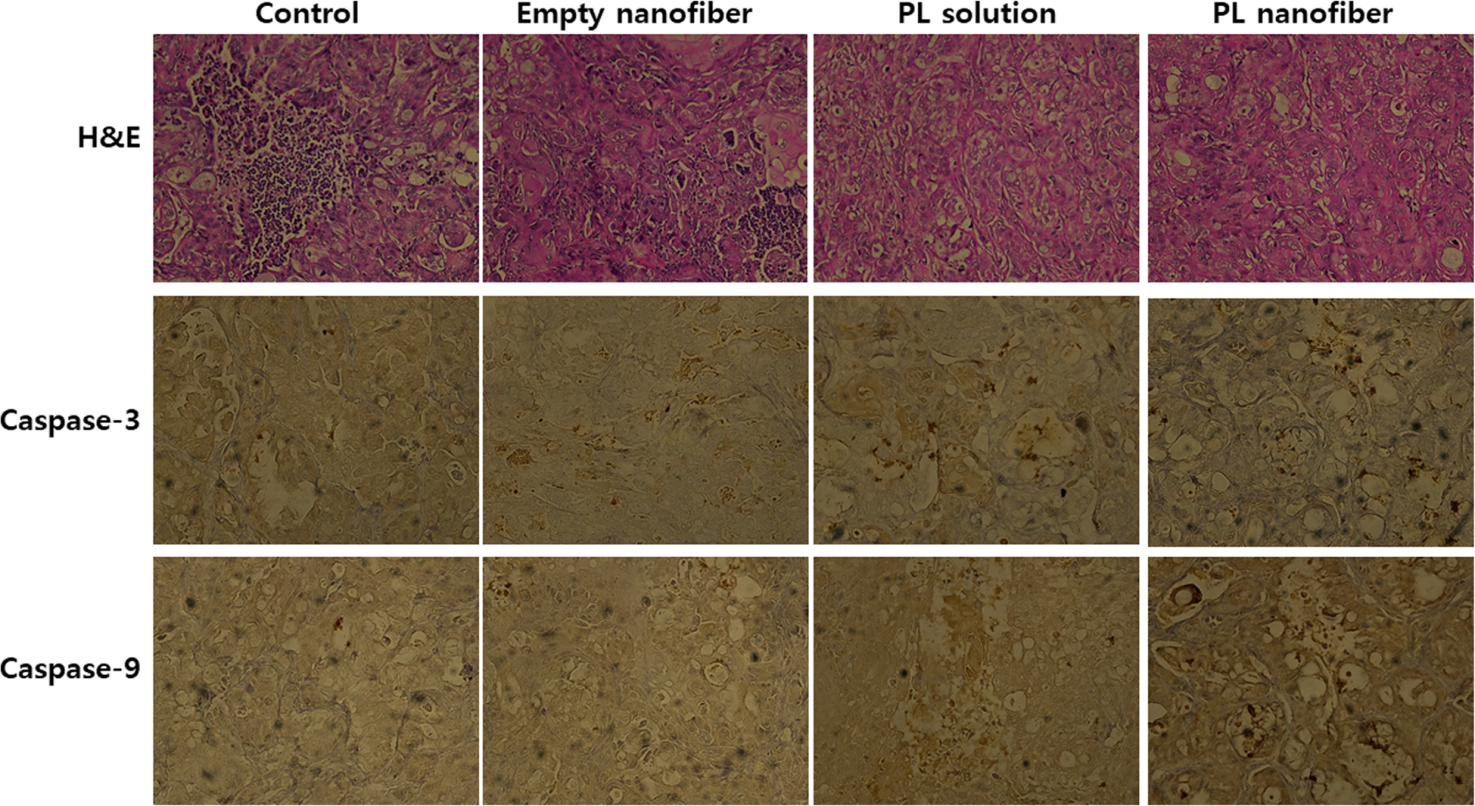
Immunohistochemical staining (× 400) of HuCC-T1 tumor tissues. Each tumor tissues were stained with BAX, caspase-3, 7, and 9, and PARP-1

HuCC-T1-bearing nude mice were prepared to assess in vivo anticancer activity of PL-incorporated nanofiber-coated stent as shown in Figs. 7 and 8. As shown in Fig. 7, the volume of HuCC-T1 tumor was gradually increased over 1 month. Growth of tumor volume in the treatment of empty nanofiber was almost similar with control treatment. PL injection properly inhibited tumor growth, compared to control treatment or empty nanofiber treatment. Especially, tumor growth was significantly inhibited by the treatment of PL-incorporated nanofiber, i.e., tumor mass in the treatment of PL-incorporated nanofiber was one third of control treatment. These results indicate that PL-incorporated nanofiber mats have superior potential in inhibition of tumor growth of CCA cells. Furthermore, the expression of caspase-3 and 9 was also increased in tumor tissues as shown in Fig. 8, indicating that released PL from nanofiber mats properly inhibited the growth of the tumor and induced apoptosis of tumor cells. Also, PL-incorporated nanofiber-coated stent has potential to inhibit CCA cells in vitro and in vivo.

## Discussion

Abnormal accumulation of ROS in tumor epithelial cells induces carcinogenesis and affects surrounding cells or tissues which constitute tumor microenvironment [39]. Then, abnormal tumor microenvironment is the key player in proliferation, migration, angiogenesis, and metastasis of cancer cells [39–41]. An elevated level of ROS in tumor microenvironment induces oxidative stress and causes DNA damage [39]. Also, ROS is also correlated with cholangiocellular proliferation and oncogenic transformation [42]. Paradoxically, increased accumulation of intracellular ROS over toxic level induces apoptosis of cancer cells and tumor suppression [39–42]. For example, Thanee et al. reported that sulfasalazine as a cystine-glutamate transporter-target drug increased intracellular ROS level and then induced cell death [43]. They also argued that therapeutic efficacy of anticancer drug can be improved by blocking the mechanism of the cell’s ROS defensive system. Also, many research groups investigated ROS-producing small molecules such as melatonin, luteolin, chloroqine, and PL to suppress CCA growth rate by increasing intracellular ROS [44–47]. Thongsom et al. reported that PL stimulates ROS accumulation in CCA cells and induces cell death by activation of caspase-3 and PARP [47]. We also observed that PL increases the accumulation of intracellular ROS in various CCA cells as shown in Fig. 5. Increased ROS level induced apoptosis signals such as BAX, caspase-3, 7, and 9, and PARP (Fig. 6). The ROS-producing capability of piperlongumine was slightly decreased on day 5 and 15 as shown in Fig. 5. These results might be due to that piperlongumine is unstable in physiological solution, and then ROS-producing capability might have been slightly decreased. Additionally, physicochemical properties of piperlongumine may be affected during the fabrication process of the nanofiber. However, our results showed that ROS-producing capacity of piperlongumine was still maintained during the 15 days of drug release experiment.

Local treatment can be applied for patients with an advanced stage or unresectable stage of CCA [48]. Among various treatment options, DES is a promising candidate for unresectable CCA patients. However, conventional DES for GI tract has no tumor-targetable drug release function, and cytotoxic agent can be eluted in all areas of polymer membrane on the stent. Chemical, physical, or biological stimuli have been applied to induce altered drug delivery in the local region [49–52]. For example, Wang et al. used a magnitude of applied tensile strain to control drug release rate on the esophageal stent, i.e., increased drug release in a specific region was observed by propagating patterned crack of multilayered coating on the stent [49]. Thin film or nanoporous devices having stimuli-responsiveness such as pH and ionic strength were also investigated for application in DES [50, 51]. Chen and Huang reported that chitosan/poly(vinyl alcohol) hybrid nanofiber membrane was crosslinked with ally disulfide to endow reductant-responsiveness, and then hybrid nanofiber membrane showed favorable biological/material features [52]. We synthesized LEse deblock copolymer and fabricate redox-responsive nanofiber mats for local application in CCA tumors. PCL/LEse-blended nanofiber mats showed increased drug release behavior with responsiveness against H_2_O_2_, indicating that drug release kinetics can be controlled by ROS level in cancer cells or tumor tissues. Furthermore, ROS-dependent drug release from nanofiber mats can be accelerated in tumor since PL is a ROS-producing agent. PL in the tumor may synergistically increase ROS level and then accelerate drug release from nanofiber mats. After all, ROS-dependent release of PL from nanofiber-coated stent synergistically inhibits CCA cells in vitro and in vivo.

## Conclusion

We fabricated ROS-sensitive nanofiber mats-coated GI stent using PCL/LEse block copolymer blend. PL was incorporated in nanofiber mats by electrospinning technique. PL release from nanofiber mats was accelerated by addition of H_2_O_2_, indicating that PL-incorporated nanofiber membranes have ROS-responsiveness. PL released from nanofiber mats at 5 days and 15 days showed appropriate anticancer activity even though its anticancer activity was slightly decreased compared to PL itself. As well as PL itself, PL released from nanofiber mats induced ROS generation and apoptosis of CCA cells. Furthermore, PL-incorporated nanofiber mats properly inhibited the growth of HuCC-T1 tumor in mice. We suggest PL-incorporated nanofiber mats prepared by PCL/LEse block copolymer blend as a promising candidate for local treatment of CCA cells.
